# Anabolic steroids among resistance training practitioners

**DOI:** 10.1371/journal.pone.0223384

**Published:** 2019-10-16

**Authors:** Ericson Pereira, Samuel Jorge Moyses, Sérgio Aparecido Ignácio, Daniel Komarchewski Mendes, Diego Sgarbi da Silva, Everdan Carneiro, Ana Maria Trindade Grégio Hardy, Edvaldo Antônio Ribeiro Rosa, Patrícia Vida Cassi Bettega, Aline Cristina Batista Rodrigues Johann

**Affiliations:** 1 Department of Physical Education, Graduate Program, Life Sciences School, Pontifícia Universidade Católica do Paraná, Curitiba, Paraná, Brazil; 2 Department of Dentistry, Graduate Program, Life Sciences School, Pontifícia Universidade Católica do Paraná, Curitiba, Paraná, Brazil; 3 Department of Pharmacology, College of Pharmacy and Pharmaceutical Sciences, The University of Toledo, Toledo, Ohio, United States of America; Universidade Federal de Juiz de Fora, BRAZIL

## Abstract

**Objective:**

To compare the use of anabolic steroids (AS), the motivation to use them, their side effects, the source of information and the form in which AS were obtained, the medical follow-up, and the periodic examinations in resistance training practitioners who are either current or former users of AS.

**Methods:**

A prevalence survey was performed in the gyms of the city of Curitiba, including 719 current and former AS users who self-administered a questionnaire. The chi-square and z of proportions (p <0.05) statistical tests were conducted.

**Results:**

Esthetics was the main motivation associated with AS intake, leading to satisfactory results. The information about the form in which to use AS was provided by doctors and AS were either purchased at the pharmacy with a prescription or illegally. Current users reported a higher number of cycles and doses, a longer duration of use, as well as larger economical investments into AS. This shows a higher consumption of such drugs, regardless of the medical follow-up and post-cycle therapy.

**Conclusion:**

Given that a change in the usage pattern was observed when increasing the AS consumption, this should be considered in the elaboration of public policies to inhibit such a trend.

## Introduction

Anabolic steroids (AS), including testosterone [1] are hormones that are usually used in a therapeutic setting [2–4]. However, they have also been illicitly employed as performance enhancers by professional competitors, school and amateur athletes [5], as well as by college students seeking improved Esthetics [5,6].

The AS medications can be administered either orally or intramuscularly [7] and their periods of use are denominated as cycles. Each cycle can range from 6 to 12 weeks, during which more than one AS administration is usually reported. The pyramid is one of the most common ways of performing a cycle. Specifically, while a gradual increase in the dosage occurs to ensure the adaptation of the body to the high doses, a gradual reduction follows to allow the recovery of the body [8].

The main testosterone drugs used as AS are as follows: Durateston®, Decadurabolin®, Winstrol® and Landerlan® [9–14].

Although the use of AS results in positive effects on performance, such as improvements in both strength and muscle mass [15], their use is also associated with changes in the anxiety and aggression patterns [6]. In addition, depression, personality and mood changes, sleep problems, irritability, and withdrawal symptoms are also common [8].Furthermore, while women may experience menstrual irregularities, clitoris hypertrophy, uterine and breasts atrophy, men may present a decrease in reproductive hormones, testicular atrophy, impotence, and gynecomastia [16].

Similarly, AS may cause acne, stretch marks, hair growth, voice alterations, pain, and abscesses after the application of injectable AS, liver changes (e.g., cholestasis, adenoma, and carcinoma), and cardiovascular events (e.g., hypertension, thrombosis, arrhythmias, systolic and diastolic dysfunction, left ventricular hypertrophy, and myocardial infarction) [8]. Moreover, the risk of sudden and unexpected death may also increase with AS usage [17, 18]. In addition, although the hospitalizations related to the use of AS are relatively low in Brazil, 1319 hospitalizations (age range: from 15 to 29 years old) were counted between 2000 and 2010, representing a burden for the health system. This number may be even higher given the failures seen in the hospitalization process [19].

Therefore, in addition to the sporting environment, the use of AS represents a problem also for the public health, considering the indiscriminate and non-therapeutic use of such drugs. The present study aims at comparing the use of AS, the motivation to use them, their side effects, the source of information and the form in which AS were obtained, the medical follow-up, and the periodic examinations in weight training practitioners who are either current or former users of AS. Given the possible side effects of the abuse of such drugs, the characterization of the AS form of use presented here is necessary. Furthermore, our results may be important for the foundation of public policies focused on informing and monitoring this publicly.

## Materials and methods

An observational cross-sectional prevalence survey was conducted. The project was approved by the Research Ethics Committee of the Pontifical Catholic University of Paraná (PUCPR)—Curitiba—Paraná - Brazil, opinion no. 1,524,203 / 2016. The study met the ethical standards of Harriss et al., 2018 [20].

**Fig 1:**
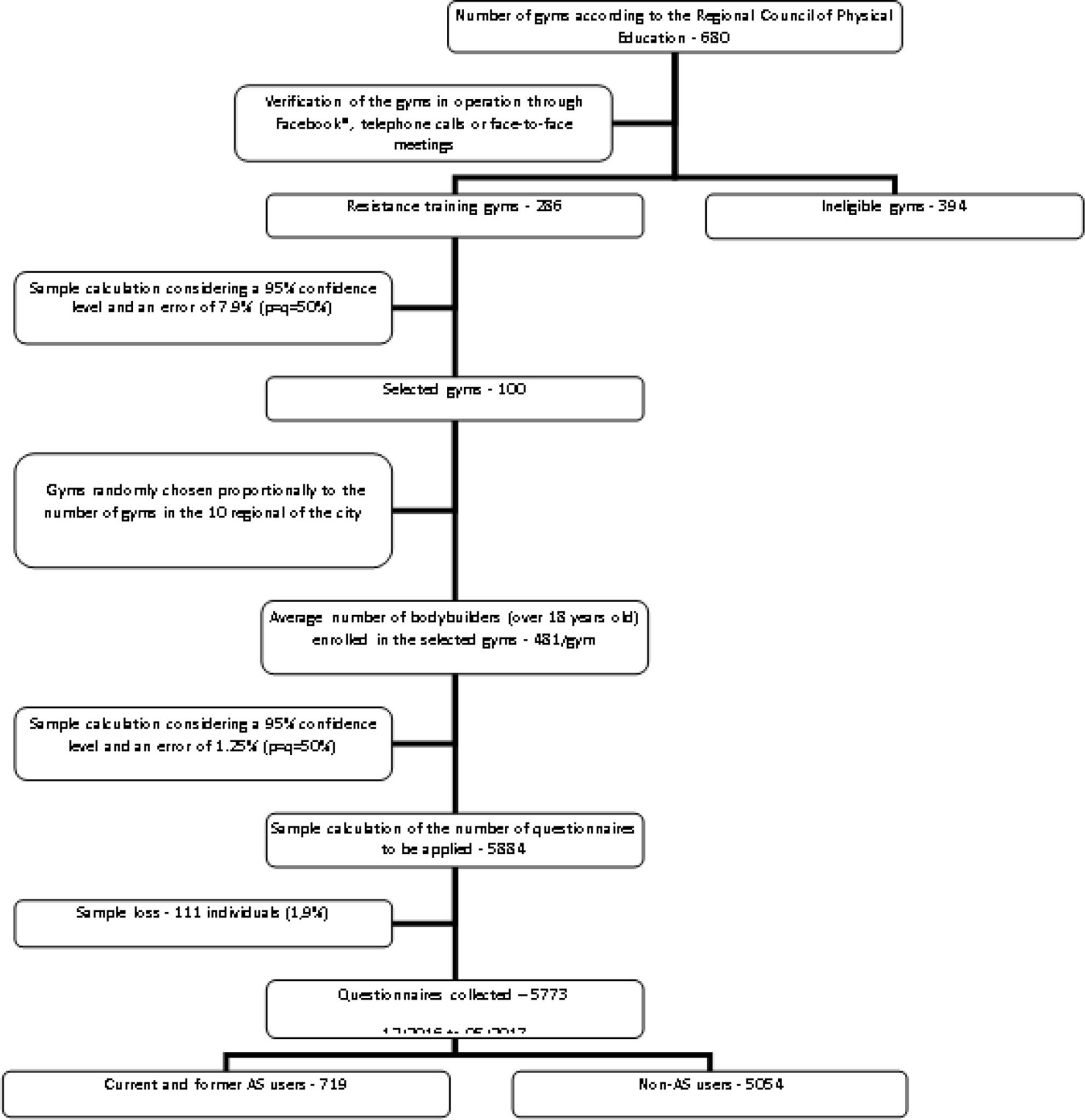
Sampling plan.

Specifically, the survey was conducted in the city of Curitiba (Brazil), which has approximately 1.9 million inhabitants and a human development index (HDI) of 0.823 [21]. Fig 1 shows the design of the sampling plan, which includes an initial survey of the number of gyms registered with the Regional Council of Physical Education, i.e., 680 gyms. Successively, only the resistance training centers were selected, leading to a total of 286 gyms. A sample calculation was performed to determine the number of gyms required in this study (considering p = q = 50%), which resulted to be 100 (given an error of 7.9% and a confidence level of 95%). Successively, the average number of students (over 18 years old) enrolled in these gyms was identified, i.e., 481 practitioners. Thereafter, a new sample calculation was performed, considering an error of 1.25% and a 95% confidence level. The survey was applied out to individuals upon entering each gym. The participants signed a disclaimer that explained informed consent, confidentiality, and anonymity for subjects. A sample of 5884 individuals was obtained. Data were collected between December 2016 and May 2017, in a manner proportional to the number of practitioners present in each gym. Finally, after the exclusion of those individuals who did not use AS, a sample of 719 current and former users of AS resulted.

The questionnaire (S1 and S2 File) was self-administered by participants using the KoboCollect application (KoboCollect, Cambridge, Massachusetts, United States) on the Samsung Tablet, Tab 2 model (Samsung, Campinas, Brazil). This questionnaire was developed for this research and validated through the clarity, construct and content indices. The aspects of construction and content were validated by health professionals, while the clarity aspect was validated with individuals of the same class, age and lifestyle of the individuals who would be researched. A pilot study was conducted that the questionnaire could be used with the intended population. The questionnaire briefly, it comprised a first part to be answered by all the resistance training practitioners participating in this research and a second part to be answered only by current and former users of AS. This second part was analyzed in the present study, as it contained questions that addressed the age at AS use onset, the number of cycles performed, the cycle length, the weekly dosage, the type of AS used, the money invested in AS, AS used, the knowledge about the post-cycle therapy (PCT), the motivation to use AS, the satisfaction with the results, the source of information about AS, the form in which AS were obtained, the medical follow-up, the periodic exams and the changes in such exams.

Successively, all the data were transferred from the Kobocollect application (KoboCollect, Cambridge, Massachusetts) to the Excel (Microsoft, Redmond, Washington) and IBM SPSS 20.0 (IBM SPSS, Armonk, New York) software. The exploratory descriptive statistics of the frequency distribution and the percentages were performed using the results presented in the tables. With regards to the inferential analyzes, the chi-square test and the z-test of proportions were conducted, in addition to the student's t-test to compare the means (significant difference at p <0.05).

## Results

In the sample studied, 73% of the practitioners were former users of AS, while 27% were current users (men: 77.7%; women: 22.3%). However, when comparing the former and current users, differences between men and women were not observed (p = 0.09). Furthermore, the mean age of the participants was similar between men (30.4 ± 7.0 years) and women (30.8 ± 7.4 years) (p = 0.57), also when considering both former (30.8 ± 7.0 years) and current (29.8 ± 7.2 years) users (p = 0.08).

The majority of the current and former users began to use AS between their 18 and 29 years of age (73.1%), with 6.7% of them starting before their 18 years of age. Specifically, the mean age at onset among the former (24.8 ± 6.0 years) and current (25.0 ± 6.7 years) users was similar (p = 0.79).

The highest percentage of the former users consumed AS for a period of time longer than one year (66.3%): 19.2, 17.5, 29.6, 14.5, and 19.2% used it for more than five and three years, one year, and six and three months, respectively.

Table 1 compares the current and former users of AS. While a higher percentage of former users only performed one cycle of AS, six cycles or more were reported by current users. In addition, a shorter duration of one to two months per cycle was mainly observed in former users, whereas cycles with a duration longer than five months were mostly found among current users. Moreover, while a dosage of 100 mg per week was commonly reported by former users, dosages higher than 301 mg per week were described by current users. In addition, injectable AS were mostly employed by current users. Furthermore, a higher percentage of former users invested up to US$ 134 for buying AS, whereas values over US$ 134 were described by current users. However, the use of AS related to esthetic reasons and curiosity was higher among former users. Specifically, of those who were curious, 60.1% only performed one cycle (p = 0.029). In contrast, the use of AS for bodybuilding was greater in current users. Of the satisfied individuals, there was a higher percentage of current users.

**Table 1:** Comparison of the number and duration of AS cycles, the dosage and type of AS used, the money invested, AS used, the motivation for the use of AS, and the subsequent satisfaction between former and current users of AS; Curitiba, 2016/2017.

Variables	Total n (%)	Former Users n (%)–n M–n F	Current Users n (%)–n M–n F	P
**Number of Cycles**				
**Only 1**	331 (46%)	284_a_ (54,1%)– 208–76	47_b_ (24,2%)– 35–12	0,0001
**2 to 5**	281 (39,1%)	199_a_ (37,9%)– 154–45	82_a_ (42,3%)– 62–20	
**6 or more**	107 (14,9%)	42_a_ (8,0%)– 38–4	65_b_ (33,5%)– 62–3	
**Duration of the cycle**				
**1 to 2 months**	376 (52,3%)	315_a_ (60,0%)– 242–73	61_b_ (31,4%)– 47–14	0,0001
**3 to 4 months**	189 (26,3%)	128_a_ (24,4%)– 100–28	61_a_ (31,4%)– 48–13	
**5 to 6 months**	58 (8,1%)	33_a_ (6,3%)– 27–6	25_b_ (12,9%) 22–3	
**7 to 12 months**	36 (5,0%)	14_a_ (2,7%)– 10–4	22_b_ (11,3%)– 18–4	
**More than 12 months**	60 (8,3%)	35_a_ (6,7%) 21–14	25_b_ (12,9%) 24–1	
**Dosage per week**				
**Unknown**	176 (24,5%)	149_a_ (28,4%)– 110–39	27_b_ (13,9%)– 20–7	0,0001
**Up to 100 mg**	296 (41,2%)	241_a_ (45,9%)– 178–73	55_b_ (28,4%)– 32–23	
**101 mg to 300 mg**	108 (15,0%)	76_a_ (14,5%)– 67–9	32_a_ (16,6%)– 28–4	
**301 mg to 500 mg**	64 (8,9%)	34_a_ (6,5%)– 31–3	30_b_ (15,5%)– 30–0	
**501 mg to 700 mg**	22 (3,1%)	8_a_ (1,5%)– 8–0	14_b_ (7,2%)– 14–0	
**Above 700 mg**	53 (7,4%)	17_a_ (3,2%)– 16–1	36_b_ (18,6%)– 35–1	
**Type**				
**Injectable**	566 (78,7%)	400_a_ (76,2%)– 339–61	166_b_ (85,6%)– 140–26	0,006
**Oral**	427 (59,4%)	301_a_ (57,3%)– 204–97	126_a_ (64,9%)– 100–26	0,065
**Other**	16 (2,2%)	11_a_ (2,1%)– 7–4	5_a_ (2,6%)– 3–2	0,697
**Value invested**				
**Up to US$ 134**	362 (56,3%)	299_a_ (64,6%)– 207–92	63_b_ (35,0%)– 46–17	0,0001
**US$ 135 to US$ 269**	139 (21,6%)	86_a_ (18,6%)– 78–8	53_b_ (29,4%)– 43–10	
**More than US$ 270**	142 (22,1%)	78_a_ (16,8%)– 65–3	64_b_ (35,6%)– 60–4	
**Motivation**				
**Esthetic**	538 (74,8%)	406_a_ (77,3%)– 302–104	132_b_ (68,0%)– 109–23	0,011
**Sports Performance**	262 (36,4%)	185_a_ (35,2%)– 155–30	77_a_ (39,7%)– 67–10	0,271
**Bodybuilding**	131 (18,2%)	56_a_ (10,7%)– 43–13	75_b_ (38,7%)– 65–10	0,0001
**Curiosity**	85 (11,8%)	75_a_ (14,3%)– 64–11	10_b_ (5,2%)– 8–2	0,001
**Therapeutic**	18 (2,5%)	12_a_ (2,3%)– 10–2	6_a_ (3,1%)– 6–0	0,539
**Other**	10 (1,4%)	7_a_ (1,3%)– 6–1	3_a_ (1,5%)– 2–1	0,829
**Satisfaction after use**				
**Yes**	613 (85,3%)	428_a_ (81,5%)– 329–99	185_b_ (95,4%)– 151–34	0,0001
**No**	106 (14,7%)	97_a_ (18,5%)– 71–26	9_b_ (4,6%)– 8–1	
**AS used**				
**Stanozolol**	436 (60,6%)	308_a_ (58,7%)– 258–50	128_a_ (66,0%)– 112–16	0,075
**Phenylpropionate, isocapronate, propionate and decanoate testosterone**	332 (46,2%)	217_a_ (41,3%)– 214–3	115_b_ (59,3%)– 112–3	0,0001
**Oxandrolone**	329 (45,8%)	214_a_ (40,8%)– 127–87	115_b_ (59,3%)– 86–29	0,0001
**Nandrolone decanoate**	235 (32,7%)	142_a_ (27,0%)– 136–6	93_b_ (47,9%)– 87–6	0,0001
**Methandrostenolone**	165 (22,9%)	89_a_ (17,0%)– 84–5	76_b_ (39,2%)– 76–0	0,0001
**Testosterone cypionate**	163 (22,7%)	85_a_ (16,2%)– 84–1	78_b_ (40,2%)– 77–1	0,0001
**Trembolone**	162 (22,5%)	75_a_ (14,3%)– 70–5	87_b_ (44,8%)– 84–3	0,0001
**Boldenone Undecylate**	129 (17,9%)	51_a_ (9,7%)– 40–11	78_b_ (40,2%)– 68–10	0,0001
**Drostanolone propionate**	109 (15,2%)	46_a_ (8,8%)– 37–9	63_b_ (32,5%)– 58–5	0,0001
**Oxymethalone**	94 (13,1%)	59_a_ (11,2%)– 57–2	35_b_ (18,0%)– 34–1	0,016
**Other**	43 (6,0%)	30_a_ (5,7%)– 20–10	13_a_ (6,7%)– 9–4	0,620

Proportion z test. The different letters in the lines indicate significant differences (p <0.05). US$: American dollar (exchange of 10/31/2018). n M: number of male. n F: number of female.

Winstrol® (stanozolol) was the most commonly used AS by both current and former users. However, a greater diversity of AS was consumed by current users, including Durateston® (phenylpropionate, isocaproate, propionate and decanoate testosterone), Landerlan® (oxandrolone), Deca-Durabolin® (nandrolone decanoate), Dianabol® (methandrostenolone), Deposteron® testosterone), Parabolan® (trenbolone), Boldenone® (boldenone undecylated), Masteron® (drostanolone propionate), and Hemogenin® (oximethalone).

Table 2 shows the presence of collateral symptoms in both current and former users, as well as their disappearance after the end of the cycle. A higher percentage of the following symptoms was observed among current users: increased libido, acne appearance, irritability/aggressiveness, hypertension, depression, and dependence.

**Table 2:** Presence of collateral symptoms in both current and former users, as well as their disappearance after the end of the cycle.

Variables	Total n (%)	Former Users n (%)	Current Users n (%)	P
**Collateral Symptoms**				
**Yes**	643 (89,4%)	466_a_ (88,8%)	177_a_ (91,2%)	0,338
**No**	76 (10,6%)	59_a_ (11,2%)	17_a_ (8,8%)	
**Symptoms**				
**Increased Libido**	327 (45,5%)	213_a_ (40,6%)	114_b_ (58,8%)	0,0001
**Acne**	308 (42,8%)	213_a_ (40,6%)	95_b_ (49,0%)	0,043
**Irritability / Aggressiveness**	233 (32,4%)	157_a_ (29,9%)	76_b_ (39,2%)	0,018
**Headache**	159 (22,1%)	119_a_ (22,7%)	40_a_ (20,6%)	0,557
**Decreased Libido**	111 (15,4%)	77_a_ (14,7%)	34_a_ (17,5%)	0,346
**Gynecomastia**	97 (13,5%)	63_a_ (12,0%)	34_a_ (17,5%)	0,054
**Hypertension**	91 (12,7%)	55_a_ (10,5%)	36_b_ (18,6%)	0,004
**Change in Menstrual Cycle**	73 (10,2%)	58_a_ (11,0%)	15_a_ (7,7%)	0,191
**Deepening of the Voice**	71 (9,9%)	50_a_ (9,5%)	21_a_ (10,8%)	0,604
**Depression**	50 (7,0%)	26_a_ (5,0%)	24_b_ (12,4%)	0,001
**Dependency**	31 (4,3%)	16_a_ (3,0%)	15_b_ (7,7%)	0,006
**Vomiting / Nausea**	25 (3,5%)	17_a_ (3,2%)	8_a_ (4,1%)	0,545
**Other**	59 (8,2%)	48_a_ (9,1%)	11_a_ (5,7%)	0,132
**Symptoms Disappeared After Cycle**				
**Yes**	555 (77,2%)	415_a_ (79,0%)	140_a_ (72,2%)	0,025
**No**	98 (13,6%)	71_a_ (13,5%)	27_a_ (13,9%)	
**Some**	66 (9,2%)	39_a_ (7,4%)	27_b_ (13,9%)	

Proportion z test. The different letters in the lines indicate significant differences (p <0.05).

Table 3 illustrates that a higher percentage of current users obtained the information related to AS from doctors and nutritionists. In addition, a higher percentage of current users received AS either through a prescription in the pharmacy or in other ways, including the black market or imports. Furthermore, a higher percentage of current users reported medical follow-ups and periodic exams (e.g., total testosterone dosage) given their usage of AS. Moreover, changes in the results of such tests were identified, mainly among the current users. Finally, a higher percentage of current users was aware of the PCT and performed it similarly to previous users.

**Table 3:** Comparison of the source of information related to AS, the way in which the AS was obtained, the medical follow-up and exams, alterations in such exams, and the knowledge and conduction of the PCT between former and current users of AS; Curitiba, 2016/2017.

Variables	Total n (%)	Former Users n (%)	Current Users n (%)	P
**Information Source**				
**Doctors**	331 (46,0%)	214_a_ (40,8%)	117_b_ (60,3%)	0,0001
**Friends**	254 (35,3%)	193_a_ (36,8%)	61_a_ (31,4%)	0,185
**Coaches**	226 (31,4%)	156_a_ (29,7%)	70_a_ (36,1%)	0,103
**Internet**	155 (21,6%)	119_a_ (22,7%)	36_a_ (18,6%)	0,234
**Nutritionist**	109 (15,2%)	68_a_ (13,0%)	41_b_ (21,1%)	0,007
**Other**	34 (4,7%)	25_a_ (4,8%)	9_a_ (4,6%)	0,945
**Way of obtaining the AS**				
**Friends**	394 (54,8%)	291_a_ (55,4%)	103_a_ (53,1%)	0,576
**Pharmacy with prescription**	271 (37,3%)	183_a_ (34,9%)	88_b_ (45,4%)	0,010
**Pharmacy without prescription**	77 (10,7%)	55_a_ (10,5%)	22_a_ (11,3%)	0,739
**Other**	119 (16,6%)	77_a_ (14,7%)	42_b_ (21,6%)	0,025
**Medical follow-up**				
**Yes**	281 (39,1%)	157_a_ (29,9%)	124_b_ (63,9%)	0,0001
**No**	438 (60,9%)	368_a_ (70,1%)	70_b_ (36,1%)	
**Conducting examinations**				
**Yes**	521 (72,5%)	358_a_ (68,2%)	163_b_ (84,0%)	0,0001
**No**	198 (27,5%)	167_a_ (31,8%)	31_b_ (16,0%)	
**Exams**				
**Total Testosterone**	402 (55,9%)	257_a_ (49,0%)	145_b_ (74,7%)	0,0001
**Cholesterol**	399 (55,5%)	272_a_ (51,8%)	127_b_ (65,5%)	0,001
**High density lipoprotein—HDL**	378 (52,6%)	254_a_ (48,4%)	124_b_ (63,9%)	0,0001
**Low density lipoprotein—LDL**	357 (36,0%)	238_a_ (45,3%)	119_b_ (61,3%)	0,0001
**Cortisol**	259 (36,0%)	161_a_ (30,7%)	98_b_ (50,5%)	0,0001
**Alanine Aminotransferase—ALT**	246 (34,2%)	146_a_ (27,8%)	100_b_ (51,5%)	0,0001
**Aspartate Aminotransferase—AST**	243 (33,8%)	146_a_ (27,8%)	97_b_ (50,0%)	0,0001
**Follicle Stimulating Hormone—** **FSH**	243 (33,8%)	147_a_ (28,0%)	96_b_ (49,5%)	0,0001
**Progesterone**	218 (30,3%)	129_a_ (24,6%)	89_b_ (45,9%)	0,0001
**Other**	68 (9,5%)	41_a_ (7,8%)	27_b_ (13,9%)	0,013
**Do not Know**	51 (7,1%)	34_a_ (6,5%)	17_a_ (8,8%)	0,289
**Exams alterations**				
**Yes**	201 (28,0%)	112_a_ (21,3%)	89_b_ (45,9%)	0,0001
**No**	518 (72,0%)	413_a_ (78,7%)	105_b_ (54,1%)	
**Knowledge PCT**				
**Yes**	567 (78,9%)	391_a_ (74,5%)	176_b_ (90,7%)	0,0001
**No**	152 (21,1%)	134_a_ (25,5%)	18_b_ (9,3%)	
**Realization PCT**				
**Yes**	288 (40,1%)	176_a_ (33,5%)	112_b_ (57,7%)	0,0001
**No**	431 (59,9%)	349_a_ (66,5%)	82_b_ (42,3%)	

Proportion z test. The different letters in the lines indicate significant differences (p <0.05).

## Discussion

One of the strengths of the present study relies on its large sample size, i.e., 719 weight training practitioners who are either current or former users of AS. Our results reveal differences between these groups, which were mainly related to the form of use: the number and duration of the AS cycles, the amount of money invested in them, the type of AS, the dosage used, and their motivation to consume them. Furthermore, differences were observed in the source of information related to the drugs, the way in which AS were obtained and the conduction of medical follow-ups.

In the current study, a higher number of former rather than current users of AS was observed, in accordance with previous literature [22, 23]. While Silva & Moreau (2003) [22] and Leifman et al., (2011) [23] separated current (17 and 5, respectively) from former (23 and 62, respectively) users into two categories, all the other surveys grouped them into a single category, making their comparison not possible. Furthermore, a larger number of current and former male users was here found, in accordance with previous literature [24, 13, 25, 26].

Practitioners’ mean age was similar between men and women, as well as between current and former users. However, although our averages are above the age ranges described in other studies [27, 10, 9], the mean corroborates with the values seen in the literature if the age at onset of AS use is considered. It is also worth mentioning that 6.7% of the individuals started the use of AS at an age younger than 18 years old, as evidenced in other studies [28–31].

Our results identified that a higher percentage of former, as opposed to current, users only conducted one AS cycle, with a shorter duration (1 to 2 months) and smaller dosages (100 mg). This strengthens the hypothesis of a curiosity-driven AS use, given that the percentage of former users who reported such a reason was also higher. In contrast, the opposite was observed in current users, who performed six or more cycles, with longer durations (5 months or more) and higher dosages (301 mg or more) than former users. While the satisfaction associated with the results can be one of the motivating hypotheses behind the regular use of AS, the practice of bodybuilding may also be an explanation to this phenomenon, since the motivation to consume AS was also greater in current users.

Injectable AS were mostly used, mainly among current users. To the best of our knowledge, this is the first study identifying the frequency of AS administration forms in this population. With regards to injectable AS, a lack of proper asepsis care during the application increases the risk of infections. As a consequence, this may lead to hospitalizations given the practitioners’ progression to abscesses, which may turn into more severe conditions, such as muscular necrosis or sepsis [32]. With regards to oral AS, high hepatoxicity is expected [33, 16].

In accordance with the literature, testosterone and stanozolol were the most commonly used AS [22, 34, 35]. Although a previous study cited testosterone as commonly used AS [33], oxandrolone, which had a high prevalence in this study, was not widely assessed in other studies.

Stanozolol was the most used AS among both groups, in accordance with Silva and Moreau (2003) [22], possibly given its popular brands that can be found both in the oral and injectable forms [36]. Similarly, nandrolone decanoate was also commonly consumed in other studies [9, 11, 22, 35, 37], possibly considering its higher accessibility (i.e., lower cost in trade) and greater disclosure [36]. Both stanozolol and nandrolone decanoate are frequently used for muscle growth, given their higher anabolic characteristics compared to androgenic AS [36].

Although more than one AS is usually used during a cycle [8], veterinary drugs, such as boldenone undecylation and trenbolone, have also been consumed [12]. A report of fulminant heart attack due to the use of boldenone in humans was reported [38]. In contrast, trenbolone may affect the liver, causing cholestatic hepatitis [39, 40] and may be associated with the proliferation of tumor cells in prostate cancers [41]. In addition, it can result in dermatitis, including severe inflammatory acne with pustules and hemorrhagic ulcerations [42].

Collateral symptoms were reported by both current and former users, as widely reported in previous literature [5, 8, 16]. However, while most symptoms disappeared after the end of the cycle, given their acuity, the chronic symptoms observed may cause slow and irreversible changes [8].

In accordance with previous studies, increased libido, acne, and irritability/aggressiveness were the main collateral symptoms reported by both current and former users [22, 9]. Increased libido may be classified as an acute side effect, given that it again decreases after the cycle [15], and it is associated with high serum levels of testosterone resulting from the use of supraphysiological dosages [5]. In contrast, considering that acne usually occurs during puberty and not in adulthood, the use of AS increases the activity of the sebaceous glands, which leads to higher oil concentrations present in the skin [8]. Furthermore, irritability/aggressiveness are often associated with the AS use [8].Although increased aggression may lead to heightened violence, such a hypothesis was not previously proven, given that the individuals involved in cases of violence also used other drugs, alcohol or had personality tendencies [24].

Transient hypertension, an acute symptom, was more frequent in current users [16]. However, this acute symptom may become chronic, since the use of AS for long periods is associated with cardiovascular diseases, including hypertension, heart attack, and stroke. The most commonly used oral AS alter the levels of lipoproteins that carry the cholesterol in the blood, increase the level of low-density lipoprotein (LDL), and decrease the high-density lipoprotein (HDL) instead [8].Therefore, their assessment is important for controlling health-related risks [22].Moreover, current users were noticed to be more cautious compared to former users, possibly because they underwent more medical follow-ups.

Dependency was also more frequent in current users. The use of AS for long periods of time may eventually affect the brain as other addictive illicit drugs, acting primarily on the dopamine, serotonin, and opioid systems [8].However, dependency may also be associated with the presence of body image disorders, such as "muscular dysmorphism", where excessive preoccupation is laid onto the musculature [24]. Moreover, the stories of success disseminated by the means of communication related to image alteration and muscular bodies motivates such body transformations [12].

A higher percentage of current users obtained information about AS from doctors. In fact, the emergence of anti-aging treatments increases the number of doctors who encourage AS therapies [43]. However, attention is drawn to the fact that doctors are prescribing such therapies to youngsters, who fail in conducting a medical follow-up, representing a big issue [13]. Therefore, the increased number of doctors prescribing the use of AS may be stimulating its use. In fact, the acquisition of such drugs in the pharmacy with a prescription was high, as described in previous literature [22, 33]. Moreover, friends, coaches, and nutritionists also influence the use of AS [44, 9]. In fact, coaches encourage their clients to consume AS for better results in the shortest time possible to improve their reputation in the academy [27].

This study did not find a definite protocol for the use of AS. Various combination, dosages, durations, and cycles were in fact used by practitioners. An empirical culture on how to best use AS according to the final objective exists, which can either be obtained from manuals or transmitted orally between users (based on their own experiences) [22]. Furthermore, this information, as well as the AS products, are widely found on the internet.

In accordance to previous studies, in addition to pharmacies, AS were found to be also illegally marketed (black market) and easy to access [33, 45, 9]. In fact, AS are marketed freely on the internet and in the gyms themselves [36]. About 1/3 of the illicitly imported products derive from Paraguay, according to the federal police of Brazil. It should be noted that these products are not regulated and that they may be falsified [46], leading to possible serious health damages.

Although most of the individuals do not conduct a medical follow-up, a higher percentage of current users performed monitoring and tests to control for the risks associated with the use of AS, including changes in vital organs (e.g., heart and liver) and dosage of testosterone. However, performing these tests does not eliminate health risks [22].

Although a great knowledge about PCT exists, a small number of individuals actually perform such a therapy. Briefly, PCT involves the use of certain medications aimed at reversing the suppression of endogenous production of testosterone at least temporarily. Its abrupt interruption, without returning to the endogenous production, can lead to a state of hypogonadism characterized by a substantial loss of muscle mass, reduced energy levels, depression, and loss of libido. However, a higher percentage of current users are performing PCT, which may be a result of the increased number of medical follow-ups conducted in this group^7^. In the present study, the following drugs used during PCT were identified: tamoxifen, human chorionic gonadotropin hormone (CGH), Clomiphene, Anastrozole, Saw Palmetto, Legalon, and Proviron. These drugs were prescribed for such a therapy in previous literature [7].

The limitations of the current study will now be highlighted. Firstly, a response bias may have affected our results, considering that the answers provided in the self-administered questionnaire depended on the participant's honesty and that confirming their veracity was not possible. In addition, as some of the information refer to a period in the past (even years before the current investigation), especially with regards to former users, uncertainties regarding the information provided may be present.

## Conclusions

Most current users performed between two to five and up to six or more cycles of AS, with a duration of five months or more and a dosage higher than 301 mg per week. They consumed the injectable type of AS, invested an amount of money higher than US$ 134 and used stanozolol. Esthetics was found to be the main reason associated with the use of such drugs and individuals were satisfied with the obtained results. Although they presented side-effects during the period of use (mainly increased libido, irritability/aggressiveness, and acne appearance), such symptoms disappeared after the use. The information related to the use of AS was mainly obtained through doctors and the drugs were purchased either through friends or at a pharmacy with a prescription. Individuals conducted medical follow-ups, had a knowledge of PCT and performed it. In contrast, most former users only conducted one cycle of AS, for a duration of one to two months, with dosages lower than 100 mg per week. They invested less than US$ 134 in AS, did not conduct medical follow-ups and did not perform PCT.

Overall, we noticed some changes in the pattern of use between former and current users of AS. Specifically, the latter reported a higher number of cycles of AS, a longer duration, increased dosage and money invested in AS, and a consequent higher and diverse consumption of AS. However, the presence of collateral symptoms does not inhibit such a consumption, possibly due to the safety provided by the medical follow-ups, which were conducted by a high number of current users. Finally, an increase in both the number of doctors providing such services and the acquisition of AS in pharmacies with a prescription was observed. This implies the need for public policies ensuring both improved information, given the symptoms and risks presented, and better control, considering that the abuse of such drugs is associated with a number of health risks.

## Supporting information

S1 File Portuguese questionnaire. (DOC)

S2 File Questionnaire. (DOC)

## References

[1] Kanayama G, Hudson JI, Pope HG Jr. Illicit anabolic–androgenic steroid use. Horm Behav 2010; 58: 111–121. 10.1016/j.yhbeh.2009.09.006 19769977 PMC2883629

[2] Carrasquilo R, Chu K, Ramasamy R. Novel Therapy for Male Hypogonadism. Curr Urol Rep 2018; 19:63 10.1007/s11934-018-0816-x 29886559

[3] Chrysant SG, Chrysant GS. Cardiovascular benefits and risks of testosterone replacement therapy in older men with low testosterone. Hosp Pract 2018; 46:47–55. 10.1080/21548331.2018.1445405 29478348

[4] Tryniszewski W, Kamiński G, Maziarz Z, Nowak M, Gadzicki M, Radek M. The assessment of testosterone and radioisotopic index of bone metabolism and bone mineral density in men with testosterone deficiency after one year of testosterone therapy. Nucl Med Rev 2018; 21:14–19. 10.5603/NMR.a2018.0002 29319133

[5] Sjöqvist F, Garle M, Rane A. Use of doping agents, particularly anabolic steroids, in sports and society. Lancet 2008; 371:1872–1882. 10.1016/S0140-6736(08)60801-6 18514731

[6] Oberlander JG, Henderson LP. The sturm und drang of anabolic steroid use: angst, anxiety, and aggression. Trends Neurosci 2012; 35:382–392. 10.1016/j.tins.2012.03.001 22516619 PMC4127319

[7] Anabolics Llewellyn W. Florida: Molecular Nutrition; 2009 10.1016/j.tins.2012.03.001

[8] Van Amsterdam J, Opperhuizen A, Hartgens F. Adverse health effects of anabolic-androgenic steroids. Regul Toxicol Pharmacol 2010; 57:117–123. 10.1016/j.yrtph.2010.02.001 20153798

[9] Silva GG, Brito AF, Nogueira FRS, Júnior JFCR, Ribeiro SLG, Oliveira CVC, Santos MAP. Prevalence of anabolic-androgenic steroids on bodybuilders of Teresina–PI. RPCD 2017; 17:115–124. [Portuguese].

[10] Nogueira FRS, Brito AF, Vieira TI, Oliveira CVC, Gouveia RLB. Prevalence of use of ergogenic aids among strength training apprentices in João Pessoa—Paraíba. Rev Bras Ciênc Esporte 2015; 37:56–64. [Portuguese].doi.org/10.1016/j.rbce.2013.12.001.

[11] Abrahin OSC, Naicha SFS, De Sousa EC, Moreira JKR, Do Nascimento VC. Prevalence of the use of anabolic androgenic steroids by physical education students and professors who wor k in health clubs. Rev Bras Med Esporte 2013; 19:27–30. [Portuguese]. 10.1590/S1517-86922013000100005

[12] Iriart JAB, Chaves JC, Orleans RG. Body cult and use of anabolic steroids by Bodybuilders. Cad Saúde Pública 2009; 25:773–782. [Portuguese]. 10.1590/s0102-311x2009000400008 19347203

[13] Santos AF, Mendonça PMH, Santos LA, Silva NF, Tavares JKL. Anabolic steroids: concepts according to muscular activity practisers in Aracaju (SE). Psicol Estud 2006; 11:371–380. [Portuguese]. 10.1590/S1413-73722006000200016

[14] Petersson A, Garle M, Holmgren P, Druid H, Krantz P, Thiblin I. Toxicological findings and manner of death in autopsied users of anabolic androgenic steroids. Drug Alcohol Depend 2006; 81:241–249. 10.1016/j.drugalcdep.2005.07.003 16137840

[15] Petersson A, Bengtsson J, Voltaire-Carlsson A, Thiblin I. Substance abusers motives for using anabolic androgenic steroids. Drug Alcohol Depend 2010; 1:170–172. 10.1016/j.drugalcdep.2010.04.008 20483546

[16] Turillazzi E, Perilli G, Di Paolo M, Neri M, Riezzo I, Fineschi V. Side Effects of AAS Abuse: An Overview. Mini-Rev Med Chem 2011; 11:374–389. 10.2174/138955711795445925 21443513

[17] Thiblin I, Mobini-Far H, Frisk M. Sudden unexpected death in a female fitness athlete, with a possible connection to the use of anabolic androgenic steroids (AAS) and ephedrine. Forensic Sci Int 2009; 184:7–11. 10.1016/j.forsciint.2008.11.004 19110387

[18] Montisci M, Mazloum RE, Cecchetto G, Terranova C, Ferrara SD, Thiene G, Basso C. Anabolic androgenic steroids abuse and cardiac death in athletes: Morphological and toxicological findings in four fatal cases. Forensic Sci Int 2012; 217:e13–e18. 10.1016/j.forsciint.2011.10.032 22047750

[19] Silva-Junior SHA. Hospital morbidity due to anabolic-androgenic steroids (AAS) consumption in Brazil. Rev Bras Med Esporte 2013; 19:108–111. [Portuguese]. 10.1590/S1517-86922013000200007

[20] Harriss DJ, Macsween A, Atkinson G. Standards for Ethics in Sport and Exercise Science Research: 2018 Update. Int J Sports Med 2017; 38:1126–1131. 10.1055/s-0043-124001 29258155

[21] IPARDES: Instituto Paranaense de Desenvolvimento Econômico e Social. Statistical notebook–municipality of Curitiba. 2018. http://www.ipardes.gov.br. (Acessed 10/25/2018). [Portuguese].

[22] Silva LSMF Moreau RLM. The use of anabolic-androgenic steroids among body builders in major gym centers in São Paulo, Brazil. Rev Bras Ciênc Farm 2003; 39:327–333. [Portuguese]. 10.1590/S1516-93322003000300012

[23] Leifman H, Rehnman C, Sjöblom E, Holgersson S. Anabolic androgenic steroids use and correlates among gym users: an assessment study using questionnaires and observations at gyms in the Stockholm region. Int J Environ Res Public Health 2011; 8:2656–2674. 10.3390/ijerph8072656 21845151 PMC3155322

[24] Kanayama G, Gruber AJ, Pope HG Jr, Borowiecki JJ, Hudson JI. Over-the-Counter Drug Use in Gymnasiums: An Underrecognized Substance Abuse Problem? Psychother Psychosom 2001; 70:137–140. 10.1159/000056238 11340414

[25] Sagoe D, Molde H, Andreassen CS, Torsheim T, Pallesen S. The global epidemiology of anabolic-androgenic steroid use: a meta-analysis and meta-regression analysis. Ann Epidemiol 2014; 24:383–398. 10.1016/j.annepidem.2014.01.009 24582699

[26] Sousa S, Rodrigues WRH, Silva RA, Zanuto EC. Profile of users anabolic steroids of city Presidente Prudente-SP. Rev Bras Nutrição Esportiva 2017; 11:383–389. [Portuguese].

[27] Al-Falasi O, Al-Dahmani K, Al-Eisaei K, Al-Ameri S, Al-Maskari F, Nagelkerke N, Schneider J. Knowledge, attitude and practice of anabolic steroids use among gym users in Al-Ain district, United Arab Emirates. Open J Sports Med 2008; 2:75–81. 10.2174/1874387000802010075

[28] Elliot DL, Cheong JW, Moe EL, Goldber L. Cross-sectional Study of Female Students Reporting Anabolic Steroid Use. Arch Pediatr Adolesc Med 2007; 161:572–77. 10.1001/archpedi.161.6.572 17548762

[29] Berning JM, Adams KJ, DeBeliso M, Stamford BA, Newman I. Anabolic androgenic steroids: use and perceived use in non-athlete college students. Educational Psychology Papers and Publications 2008; 1–19. 10.3200/JACH.56.5.499-504 18400661

[30] Tahtamouni LH, Mustafa NH, Alfaouri AA, Hassan IM, Abdalla MY, Yasin SR. Prevalence and risk factors for anabolic-androgenic steroid abuse among Jordanian collegiate students and athletes. Eur J Public Health 2008; 18: 661–665. 10.1093/eurpub/ckn062 18603598

[31] Andrade AG, Duarte PCAV, Barroso LP, Nishimura R, Alberghini DG, Oliveira LG. Use of alcohol and other drugs among Brazilian college students: effects of gender and age. Rev Bras Psiquiatr 2012; 34:294–305. 10.1016/j.rbp.2012.02.002 23429775

[32] Cardozo-Filho NS, Gaspar EF, Siqueira KL, Monteiro GC, Andreoli CV, Ejnisman B, Cohen M. Pyomyositis in ath letes after the use of anabolic steroids: case reports. Rev Bras Ortop 2011; 46:97–100. [Portuguese]. 10.1016/S2255-4971(15)30185-3 27026995 PMC4799224

[33] Maior AS, Bernasconi A, Sanches JF, Simão R, Menezes P, Miranda H, Nascimento JHM. Anabolic androgenic steroids use in two cities of Rio Grande do Sul. Rev Bras Prescrição e Fisiol do Exercício 2009; 3:580–591 [Portuguese].

[34] Frizon F, Macedo SMD, Yonamine M. Use of anabolic-androgenic steroids by sports practitioners attending the main gym centers in Erechim and Passo Fundo (Brazil). Rev Ciênc Farm Básica Apl 2005; 26:227–232 [Portuguese].

[35] Silva PRP, Machado Júnior LC, Figreiredo VC, Cioffi AP, Prestes MC, Czepielewski MA. Prevalence of the Use of Anabolic Agents among Strength Training Apprentices in Porto Alegre, RS. Arq Bras Endocrinol Metab 2007; 51:104–110 [Portuguese]. 10.1590/S0004-27302007000100017 17435863

[36] Nogueira FRS, Souza AA, Brito AF. Prevalence of the use and ergogenic resources effects by body builders in Brazilian academies: a systematic review. Rev Bras Ativ Fis Saúde 2013; 18:16–30 [Portuguese].

[37] Lood Y, Eklund A, Garle M, Ahlner J. Anabolic androgenic steroids in police cases in Sweden 1999–2009. Forensic Sci Int 2012; 219:199–204. 10.1016/j.forsciint.2012.01.004 22269132

[38] White M, Brennan E, Ren KYM, Shi M, Thakrar A. Anabolic Androgenic Steroid Use as a Cause of Fulminant Heart Failure. Can J Cardiol 2018; 34: 1–3. 10.1016/j.cjca.2018.06.008 30205989

[39] Anand JS, Chodorowski Z, Hajduk A, Waldman W. Cholestasis Induced by Parabolan Successfully Treated with the Molecular Adsorbent Recirculating System. ASAIO J 2006; 52:117–118. 10.1097/01.mat.0000196712.32953.21 16436902

[40] Boks MN, Tiebosch AT, van der Waaij LA. A jaundiced bodybuilder cholestatic hepatitis as side effect of injectable anabolic-androgenic steroids. J Sports Sci 2017; 35:2262–2264. 10.1080/02640414.2016.1265659 27937337

[41] Lee HS, Jung DW, Han S, Kang HS, Suh JH, Oh HS, Hwang MS, Moon G, Park Y, Hong JH, Koo YE. Veterinary drug, 17b-trenbolone promotes the proliferation of human prostate cancer cell line through the Akt/AR signaling pathway. Chemosphere 2018; 198:364–369. 10.1016/j.chemosphere.2018.01.145 29421751

[42] Kraus SL, Emmert S, Schon MP, Haenssle HA. The dark side of beauty: acne fulminans induced by anabolic steroids in a male bodybuilder. Arch Dermatol 2012; 148:1210–1212.2 10.1001/archdermatol.2012.855 23069972

[43] Moraes DR, Castiel LD, Ribeiro APPGA. “No” for stacked young bodybuilders, “yes” for manthers: the biomedical discourse on anabolic steroids and health. Cad Saúde Pública 2015; 31:1131–1140 [Portuguese]. 10.1590/0102-311X00068914 26200362

[44] Araújo LR, Andreolo J, Silva MS. Use of alimentary supplement and anabolizante for apprentices of muscular activity in the academies of Goiânia-GO. Rev Bras Ciên e Mov 2002; 10:13–18 [Portuguese].

[45] Kimergard A, McVeigh J. Environments, risk and health harms: a qualitative investigation into the illicit use of anabolic steroids among people using harm reduction services in the UK. BMJ Open 2014; 4:1–7. doi: 10.1136/10.1136/bmjopen-2014-005275 PMC4054627 24898090

[46] Neves DBJ, Marcheti RGA, Caldas ED. Incidence of anabolic steroid counterfeiting in Brazil. Forensic Sci Int 2013; 228:81–83. 10.1016/j 23522522

