# Correction: Anabolic steroids among resistance training practitioners

**DOI:** 10.1371/journal.pone.0226208

**Published:** 2019-12-09

**Authors:** Ericson Pereira, Samuel Jorge Moyses, Sérgio Aparecido Ignácio, Daniel Komarchewski Mendes, Diego Sgarbi da Silva, Everdan Carneiro, Ana Maria Trindade Grégio Hardy, Edvaldo Antônio Ribeiro Rosa, Patrícia Vida Cassi Bettega, Aline Cristina Batista Rodrigues Johann

There is an error in affiliation 3 for author Ana Maria Trindade Grégio Hardy. The correct affiliation 3 is: Department of Physiology and Pharmacology, College of Medicine and Life Sciences, University of Toledo, Ohio, United States of America.
