# Supplementary material for: Anabolic steroids among resistance training practitioners
Source: PLoS One. 2019 Oct 16;14(10):e0223384. doi: 10.1371/journal.pone.0223384 (PMC6795452; doi:10.1371/journal.pone.0223384)
Supplement: S1 File — (DOC) [file pone.0223384.s001.doc]

## Questionário:

Questionário nº:__________

**Favor preencher esse questionário. Após o término, deposite-o na urna. As informações fornecidas serão mantidas em sigilo.**

1. Informe:
2. Sexo: ( ) Masculino ( ) Feminino
3. Idade: ____________ anos
4. Peso: ________________ kg
5. Altura: ________________ m
6. Profissão: _________________
7. Estado Civil:
   1. ( ) Solteiro
   2. ( ) Casado
   3. ( ) Divorciado/Separado
   4. ( ) Viúvo(a)
8. Escolaridade:
   1. ( ) Ensino Fundamental
   2. ( ) Ensino Médio Incompleto
   3. ( ) Ensino Médio Completo
   4. ( ) Ensino Superior Incompleto
   5. ( ) Ensino Superior Completo
9. A quanto tempo você pratica musculação?
   1. ( ) Menos de 6 meses
   2. ( ) 6 meses a 1 ano
   3. ( ) 1 ano a 3 anos
   4. ( ) Mais de 3 anos
10. Qual o período costuma treinar normalmente?
    1. ( ) Manhã b. ( ) Tarde c. ( ) Noite
11. Pratica outra atividade além da musculação?
    1. ( ) Sim. Qual ou Quais?______________________________________
    2. ( ) Não
12. Qual a frequência que você treina musculação?
    1. ( ) 2 vezes por semana
    2. ( ) 3 vezes por semana
    3. ( ) 4 vezes por semana
    4. ( ) 5 ou mais vezes por semana
13. Quantas horas treina musculação durante a SEMANA?
    1. ( ) Até 1h
    2. ( ) Até 3h
    3. ( ) Até 5h
    4. ( ) Mais de 5h
14. Qual o objetivo do treino realizado neste período (pode marcar mais de uma)?
15. ( ) Força
16. ( ) Hipertrofia
17. ( ) Emagrecimento
18. ( ) Resistência
19. ( ) Outro. Qual:_________________
20. Faz acompanhamento nutricional?
    1. ( ) Sim b. ( ) Não
21. Faz o uso de suplementos alimentares?
    1. ( ) Sim b. ( ) Não

Se “Sim”. Qual ou quais (pode marcar mais de uma)?

1. ( ) Proteína
2. ( ) Aminoácido
3. ( ) Maltodextrina / Dextrose
4. ( ) Pré treino
5. ( ) Outro. Qual:____________________

Se “Sim”. Quantos dias na semana?

1. ( ) Até 3 dias
2. ( ) Até 5 dias
3. ( ) Até 6 dias
4. ( ) Diariamente
5. Com relação à utilização de anabolizantes, assinale (atualmente):
6. ( ) Já utilizou no passado
7. ( ) Utiliza atualmente
8. ( ) Pensa em fazer uso no futuro
9. ( ) Não utiliza
10. ( ) Não tem conhecimento sobre o que são os anabolizantes

**Se você marcou na questão “12” a letra:**

**“Ä” Siga para a questão 13 e continue respondendo ao questionário até o final.**

**“B” Siga para a questão 14 e continue respondendo ao questionário até o final.**

**“C” Siga para a questão 29 e continue respondendo ao questionário até o final.**

**“D ou E” Siga para a última página e responda o quadro.**

1. Quando foi a última vez que usou anabolizante?
2. ( ) Mais de 3 meses
3. ( ) Mais de 6 meses
4. ( ) Mais de 1 ano
5. ( ) Mais de 3 anos
6. ( ) Mais de 5 anos
7. Começou a utilizar com qual idade? _____________________________
8. Quantos ciclos já realizou?
   1. ( ) 1
   2. ( ) Menos de 5
   3. ( ) Menos de 10
   4. ( ) Mais de 10
9. Qual a duração do seu ciclo (Maior período que ficou sem para o uso de anabolizantes)?
   1. ( ) 1 a 2 meses
   2. ( ) 3 a 4 meses
   3. ( ) 5 a 6 meses
   4. ( ) 8 a 12 meses
   5. ( ) Mais de 12 meses
10. Sabe o que é Terapia Pós Ciclo (TPC)?
    1. ( ) Sim b. ( ) Não
11. Realiza algum protocolo de (TPC)?
    1. ( ) Sim. Qual a Duração?____________________
    2. ( ) Não
12. Caso tenha respondido “Sim” na questão 18. Quais medicamentos utiliza na TPC?

________________________________________________________________________

1. Ficou satisfeito com o resultado?
   1. ( ) Sim b. ( ) Não
2. Qual fonte de informação e/ou indicação, você usa para utilizar os anabolizantes (pode marcar mais de uma)?
3. ( ) Instrutor / Técnico / Personal Trainer
4. ( ) Amigo
5. ( ) Médico
6. ( ) Internet
7. ( ) Outro. Qual:___________________
8. Como obteve esses anabolizantes (pode marcar mais de uma)?
   1. ( ) Na Farmácia, com receita
   2. ( ) Na Farmácia, sem receita
   3. ( ) Amigos
   4. ( ) Outros.___________________
9. Durante o seu período de uso, já sentiu algum sintoma colateral?
   1. ( ) Sim b. ( ) Não

Se “Sim”. Qual ou quais (pode marcar mais de uma)?

- 1. ( ) Pressão Alta
  2. ( ) Dores de cabeça
  3. ( ) Náuseas/Vômitos
  4. ( ) Irritabilidade/Agressividade
  5. ( ) Acne/aparecimento de espinhas
  6. ( ) Dependência
  7. ( ) Depressão
  8. ( ) Engrossamento da voz
  9. ( ) Aumento da libido
  10. ( ) Diminuição a libido
  11. ( ) Amenorréia
  12. ( ) Ginecomastia
  13. ( ) Outro. _____________________

1. Após parar com o uso de anabolizantes todos os sintomas desapareceram?
   1. ( ) Sim b. ( ) Não c. ( ) Alguns. Qual ou quais? _______________
2. Possui acompanhamento médico devido ao uso de anabolizantes?
3. ( ) Sim. Qual especialidade? ____________________________________
4. ( ) Não
5. Realiza exames periódicos por causa do uso de anabolizantes?
   1. ( ) Sim b. ( ) Não
6. Quais exames costuma realizar (pode marcar mais de uma)?
   1. ( ) Testosterona total
   2. ( ) FSH
   3. ( ) Colesterol
   4. ( ) HDL
   5. ( ) Progesterôna
   6. ( ) Cortisol
   7. ( ) LDL
   8. ( ) AST
   9. ( ) ALT
   10. ( ) Outro. Qual ou Quais? _________________
7. Observou alteração em algum deles?
   1. ( ) Não b. ( ) Sim. Qual ou Quais? _______________________
8. Qual ou quais anabolizantes você já utilizou/utiliza ou pretende utilizar caso nunca tenha usado (pode marcar mais de um)?

| Anabolizante | | Anabolizante | |
| --- | --- | --- | --- |
| ( ) | Oxandrolona | ( ) | Trembolona |
| ( ) | Estanozolol | ( ) | Boldenona |
| ( ) | Durateston | ( ) | Nandrolona |
| ( ) | Deposteron | ( ) | Masteron |
| ( ) | Deca durabolin | ( ) | Testosterona |
| ( ) | Hemogenin | ( ) | Halotestin |
| ( ) | Dianabol | ( ) | Outro. Qual?_________________ |

1. Quanto normalmente gasta/gastou com a compra de anabolizantes por ciclo? R$ _______
2. Os anabolizantes utilizados são de uso (pode marcar mais de uma):
3. ( ) Oral b. ( ) Injetável c. ( ) Outro. Qual: _____________________
4. Qual motivo o levou a fazer uso de anabolizantes (pode marcar mais de uma)?
5. ( ) Estético d. ( ) Terapêutico
6. ( ) Desempenho esportivo e. ( ) Curiosidade
7. ( ) Fisiculturismo f. ( ) Outro. Qual:___________________

Abaixo seguem algumas perguntas sobre itens do seu domicílio para efeito de classificação econômica. Todos os itens de eletroeletrônicos citados devem estar funcionando, incluindo os que estão guardados. Caso não estejam funcionando, considere apenas se tiver intenção de consertar ou repor nos próximos seis meses.

| Item | | Quantidade que possui: | | | | |
| --- | --- | --- | --- | --- | --- | --- |
|  |  | Não Possui | 1 | 2 | 3 | 4 ou + |
| Banheiros. | |  |  |  |  |  |
| Empregados mensalistas, considerando apenas os que trabalham pelo menos cinco dias por semana. | |  |  |  |  |  |
| Automóveis de passeio exclusivamente para uso particular. | |  |  |  |  |  |
| Microcomputadores, considerando computadores de mesa, laptops, notebooks e desconsiderando tablets ou smartphones. | |  |  |  |  |  |
| Lavadora de louças. | |  |  |  |  |  |
| Geladeiras. | |  |  |  |  |  |
| Freezers independentes ou parte da geladeira duplex. | |  |  |  |  |  |
| Máquinas de lavar roupa, excluindo tanquinho. | |  |  |  |  |  |
| DVD, incluindo qualquer dispositivo que leia DVD e desconsiderando DVD de automóvel. | |  |  |  |  |  |
| Fornos de micro-ondas. | |  |  |  |  |  |
| Motocicletas, desconsiderando as usadas exclusivamente para uso profissional. | |  |  |  |  |  |
| Máquinas secadoras de roupas, considerando lava e seca. | |  |  |  |  |  |
| A água utilizada neste domicílio é proveniente de: | | | | | | |
|  | Rede geral de distribuição | | | | | |
|  | Poço ou nascente | | | | | |
|  | Outro meio | | | | | |
| Considerando o trecho da rua do seu domicílio, você diria que a rua é: | | | | | | |
|  | Asfaltada/Pavimentada | | | | | |
|  | Terra/Cascalho | | | | | |
| Qual é o grau de instrução do chefe da família? Considere como chefe da família a pessoa que contribui com a maior parte da renda do domicílio. | | | | | | |
|  | Analfabeto / Fundamental I incompleto | | | | | |
|  | Fundamental I completo / Fundamental II incompleto | | | | | |
|  | Fundamental completo/Médio incompleto | | | | | |
|  | Médio completo/Superior incompleto | | | | | |
|  | Superior completo | | | | | |
