# Supplementary material for: Anabolic steroids among resistance training practitioners
Source: PLoS One. 2019 Oct 16;14(10):e0223384. doi: 10.1371/journal.pone.0223384 (PMC6795452; doi:10.1371/journal.pone.0223384)
Supplement: S2 File — (DOC) [file pone.0223384.s002.doc]

## Questionnaire:

Questionnaire nº:__________

1. Inform:
2. Gender: ( ) Male ( ) Female
3. Age: ____________ years
4. Weight: ________________ kg
5. Height: ________________ m
6. Profession: _________________
7. Marital Status:
   1. ( ) Single
   2. ( ) Married
   3. ( ) Divorced
   4. ( ) Widower
8. Schooling:
   1. ( ) Elementary School
   2. ( ) Incomplete high school
   3. ( ) Complete high school
   4. ( ) Incomplete graduated
   5. ( ) Graduated
9. How long have you been resistance trainning?
   1. ( ) Less than 6 months
   2. ( ) 6 months to 1 year
   3. ( ) 1 year to 3 years
   4. ( ) More than 3 years
10. What period do you usually train normally?
    1. ( ) Morning b. ( ) Afternoon c. ( ) Night
11. Do you practice anything other than resistance trainning?
    1. ( ) Yes. Which?______________________________________
    2. ( ) No
12. How often do you train resistance trainning?
    1. ( ) 2 times per week
    2. ( ) 3 times a week
    3. ( ) 4 times a week
    4. ( ) 5 or more times a week
13. How many hours do you train resistance trainning during the WEEK?
    1. ( ) Until 1h
    2. ( ) Until 3h
    3. ( ) Until 5h
    4. ( ) More than 5h
14. What is the purpose of the training performed during this period (you can score more than one)?
15. ( ) Force
16. ( ) Hypertrophy
17. ( ) Weight loss
18. ( ) Endurance
19. ( ) Other. Which:_________________
20. Do you have nutritional monitoring?
    1. ( ) Yes b. ( ) No
21. Do you use dietary supplements?
    1. ( ) Yes b. ( ) No

If “YES”. Which (can mark more than one)?

1. ( ) Protein
2. ( ) Amino Acid
3. ( ) Maltodextrin / Dextrose
4. ( ) Pre workout
5. ( ) Other. Which:____________________

If “YES”. How many days a week?

1. ( ) Up to 3 days
2. ( ) Up to 5 days
3. ( ) Up to 6 days
4. ( ) Daily
5. Regarding the use of anabolic steroids, please note (currently):
6. ( ) Have you used it in the past
7. ( ) Currently uses
8. ( ) Think about making use in the future
9. ( ) Does not use
10. ( ) Not aware of what anabolic steroids are

**If you marked in question “12” the letter:**

**“A” Go to question 13 and continue answering the questionnaire until the end.**

**“B” Go to question 14 and continue answering the questionnaire until the end.**

**“C” Go to question 29 and continue answering the questionnaire until the end.**

**“D or E” Go to the last page and answer the box.**

1. When was the last time you used anabolic?
2. ( ) More than 3 months
3. ( ) More than 6 months
4. ( ) More than 1 year
5. ( ) More than 3 year
6. ( ) More than 5 year
7. Started using at what age? _____________________________
8. How many cycles have you performed?
   1. ( ) 1
   2. ( ) Less than 5
   3. ( ) Less than 10
   4. ( ) More than 10
9. How long is your cycle (Longest period without using anabolic steroids)?
   1. ( ) 1 to 2 months
   2. ( ) 3 to 4 months
   3. ( ) 5 to 6 months
   4. ( ) 8 to 12 months
   5. ( ) More than 12 months
10. Do you know what Post Cycle Therapy (PCT) is?
    1. ( ) Yes b. ( ) No
11. Do you perform any protocol (PCT)?
    1. ( ) Yes. How long?____________________
    2. ( ) No
12. If you answered “Yes” to question 18. What medicines do you use for PCT?

________________________________________________________________________

1. Was pleased with the result?
   1. ( ) Yes b. ( ) No
2. What source of information and / or indication do you use to use anabolic steroids (you can check more than one)?
3. ( ) Instructor / Personal Trainer
4. ( ) Friend
5. ( ) Doctor
6. ( ) Internet
7. ( ) Other. Which:___________________
8. How did you get these anabolic steroids (you can score more than one)?
   1. ( ) In Pharmacy, with prescription
   2. ( ) In Pharmacy, without prescription
   3. ( ) Friends
   4. ( ) Others.___________________
9. Have you experienced any side symptoms during your period of use?
   1. ( ) Yes b. ( ) No

If “YES”. Which (can mark more than one)?

- 1. ( ) High pressure
  2. ( ) Headaches
  3. ( ) Nausea / Vomiting
  4. ( ) Irritability / Aggressiveness
  5. ( ) Acne
  6. ( ) Dependency
  7. ( ) Depression
  8. ( ) Voice thickening
  9. ( ) Increased libido
  10. ( ) Decreased libido
  11. ( ) Amenorrhea
  12. ( ) Gynecomastia
  13. ( ) Other. _____________________

1. After stopping anabolic use all symptoms disappeared?
   1. ( ) Yes b. ( ) No c. ( ) Some. Which? _______________
2. Has medical advice from anabolic steroids?
3. ( ) Yes. What specialty? ____________________________________
4. ( ) No
5. Performs periodic tests for use of anabolic steroids?
   1. ( ) Yes b. ( ) No
6. What exams do you usually take (may you score more than one)?
   1. ( ) Total Testosterone
   2. ( ) FSH
   3. ( ) Colesterol
   4. ( ) HDL
   5. ( ) Progesterone
   6. ( ) Cortisol
   7. ( ) LDL
   8. ( ) AST
   9. ( ) ALT
   10. ( ) Other. Which? _________________
7. Have you noticed any changes?
   1. ( ) No b. ( ) Yes. Which? _______________________
8. Which or which anabolic steroids you have used or intend to use if you have never used them (you can check more than one)?

| anabolic steroids | | anabolic steroids | |
| --- | --- | --- | --- |
| ( ) | Oxandrolone | ( ) | Trembolone |
| ( ) | Estanozolol | ( ) | Boldenone |
| ( ) | Durateston | ( ) | Nandrolone |
| ( ) | Deposteron | ( ) | Masteron |
| ( ) | Deca durabolin | ( ) | Testosterone |
| ( ) | Hemogenin | ( ) | Halotestin |
| ( ) | Dianabol | ( ) | Other. Which?_________________ |

1. How much do you typically spend / spend on buying anabolic steroids per cycle? R$ _______
2. The anabolic steroids used are for use (may mark more than one):
3. ( ) Oral b. ( ) Injectable c. ( ) Other. Which: _____________________
4. Why did you use anabolic steroids (you can check more than one)?
5. ( ) Aesthetic d. ( ) Therapeutic
6. ( ) Sports performance e. ( ) Curiosity
7. ( ) Bodybuilding f. ( ) Other. Which:___________________

Below are some questions about household items for the purpose of economic classification. All cited electronics items must be working, including those stored. If they are not working, consider only if you intend to repair or replace within the next six months.

| Item | | Quantity you own:: | | | | |
| --- | --- | --- | --- | --- | --- | --- |
|  |  | Does not have | 1 | 2 | 3 | 4 ou + |
| Toilets. | |  |  |  |  |  |
| Monthly employees, considering only those who work at least five days a week. | |  |  |  |  |  |
| Passenger cars exclusively for private use. | |  |  |  |  |  |
| Computers, considering desktops, laptops, notebooks and disregarding tablets or smartphones. | |  |  |  |  |  |
| Dish washer. | |  |  |  |  |  |
| Refrigerators. | |  |  |  |  |  |
| Independent freezers or part of the duplex refrigerator. | |  |  |  |  |  |
| Washing machines, excluding six pack. | |  |  |  |  |  |
| DVD, including any device that reads DVD and disregarding car DVD. | |  |  |  |  |  |
| Microwave ovens. | |  |  |  |  |  |
| Motorcycles, disregarding those used exclusively for professional use. | |  |  |  |  |  |
| Clothes drying machines, considering lava and dry. | |  |  |  |  |  |
| The water used in this household comes from: | | | | | | |
|  | General distribution network | | | | | |
|  | Well or spring | | | | | |
|  | Other | | | | | |
| Considering the stretch of street from your home, you would say the street is: | | | | | | |
|  | Asphalted / Paved | | | | | |
|  | Earth / Gravel | | | | | |
| What is the educational level of the head of household? Consider as head of household the person who contributes most of the household income. | | | | | | |
|  | Illiterate / Elementary I incomplete | | | | | |
|  | Elementary I complete / Elementary II incomplete | | | | | |
|  | Elementary School Complete / high school | | | | | |
|  | high school / Graduated Incomplete | | | | | |
|  | Graduated | | | | | |
